# Variable Variation: Annual and Seasonal Changes in Offspring Sex Ratio in a Bat

**DOI:** 10.1371/journal.pone.0036344

**Published:** 2012-05-03

**Authors:** Robert M. R. Barclay

**Affiliations:** Department of Biological Sciences, University of Calgary, Calgary, Alberta, Canada; University of Western Ontario, Canada

## Abstract

Many organisms produce offspring with sex-ratios that deviate from equal numbers of males and females, and numerous adaptive explanations have been proposed. In some species, offspring sex-ratio varies across the reproductive season, again with several explanations as to why this might be adaptive. However, patterns for birds and mammals are inconsistent, and multiple factors are likely involved. Long-term studies on a variety of species may help untangle the complexity. I analyzed a long-term data set on the variation in offspring sex-ratio of the big brown bat, *Eptesicus fuscus*, a temperate-zone, insectivorous species. Sex ratio varied seasonally, but only in some years. Births early in the season were significantly female biased in years in which parturition occurred relatively early, but not in years with late parturition. Survival of female pups increased with earlier median birth date for the colony, and early-born females were more likely to survive and reproduce as one-year olds, compared to later-born pups. I argue that, due to the unusual timing of reproductive activities in male and female bats that hibernate, producing female offspring early in the year increases their probability of reproducing as one year olds, but this is not the case for male offspring. Thus, mothers that can give birth early in the year, benefit most by producing a female pup. The relative benefit of producing female or male offspring varies depending on the length of the growing season and thus the time available for female pups to reach sexual maturity. This suggests that not only does sex-ratio vary seasonally and among years, depending on the condition of the mother and the environment, but also likely varies geographically due to differences in season length.

## Introduction

Sex-allocation theory is viewed as a pillar of evolutionary theory [1]–[3], and variation in offspring sex-ratio has been extensively studied empirically (e.g. [3], [4]), and theoretically (e.g. [2], [5]–[9]). Since Fisher [5] demonstrated that investment by mothers in male and female offspring should be equal, studies on a wide variety of organisms, have shown that offspring sex-ratio varies (e.g. reviews in [4], [10]). Hypotheses to explain such variation have focussed on differences in the costs and benefits to mothers of producing sons and daughters. If the costs of producing offspring differ, equal investment should theoretically result in skewed sex ratios. Such cost differences may include differences in the size of males and females at independence [2], or differences in the costs associated with competition depending on the dispersal behaviour of offspring (Local Resource Competition) [2], [11].

Offspring sex ratio also may vary in relation to the reproductive value of offspring and the consequent fitness benefits accruing to mothers by producing sons or daughters. In particular, Trivers and Willard [6] argued that in polygynous species, mothers able to provide greater than average resources, should invest more in offspring of the sex that benefits most from the added investment. In many species, preferential investment should go to male offspring, because their size and condition influences reproductive success more so than for females. The ability to invest more in offspring may result from differences in female body condition or territory, or more generally due to variation in environmental conditions across landscapes or years. Results of published work vary in the degree to which they support predictions based on the Trivers-Willard hypothesis (e.g. [2], [4]). Numerous authors comment on the inconsistent results and complex interactions among factors in studies of birds and mammals [1]–[3], [12], [13].

In the context of the Trivers-Willard hypothesis, an increasing number of studies on amphibians [14], birds [15]–[17] and mammals [8], [13], [18]–[21], including humans [22], [23], report that offspring sex-ratio varies seasonally. Again, several hypotheses could explain the skew. For example, early birth may result in larger adult size which may influence reproductive success in one sex more than the other (essentially, Trivers-Willard). Alternatively, if the probability of early sexual maturity is differentially influenced by birth date, then early births should be biased towards the sex influenced the most [9].

While some studies report consistent patterns of sex-ratio variation across years (e.g. [18]), others found that variation in weather and the timing of reproduction among years was associated with variation in the pattern of sex ratio skew (e.g. [1], [24]). Again, this is related to the condition of females in good and bad years, and the benefits of early birth in early or late reproductive periods.

Complex interactions between maternal condition and environmental condition, and variation in both on several time scales (seasonal, annual), are thought to contribute to the confusing offspring sex-ratios in birds and mammals [1], [3], [12], [13]. Aside from considering multiple variables and their interactions, long-term studies on a variety of taxa may be key to explaining the complexities of sex allocation [2], [12].

Data from a long-term study on a population of big brown bats (*Eptesicus fuscus*) indicated that complex seasonal and annual variation in offspring sex ratio occurred. The purpose of this paper is to analyze that variation in light of sex-allocation hypotheses. In particular, I tested predictions arising from the hypothesis that offspring sex-ratio varies seasonally associated with differences in the age of sexual maturity between males and females, and the probability of early reproduction [9]. If one sex has a greater probability of reaching sexual maturity and reproducing earlier than the other, and that probability is influenced by the body size or condition attained at the end of the growing season, then I predicted that early births within a year should be skewed towards that sex.

## Materials and Methods

### Ethics Statement

Capture and handling of animals followed the Guidelines on Care and Use of Wildlife established by the Canadian Council on Animal Care. The protocol (#BI-09R-01) was approved by the University of Calgary Life and Environmental Sciences Animal Care Committee, and permits for field work were obtained from the Fish and Wildlife Division of Alberta Sustainable Resource Development.

### Study species

The big brown bat (*E. fuscus*) is an aerial insectivorous species widely distributed across much of North America. In my study area, mass of adult, non-pregnant females ranges from 15 to 20 g (unpub. data). In general, adult females are slightly larger than adult males [25]. During the summer, reproductive female *E. fuscus* congregate in maternity colonies, typically in hollow trees or buildings [26]. Litter size varies geographically, with individuals in eastern North America typically giving birth to two young, while individuals in western populations generally have single offspring [26]–[29]. In my study area, approximately 90% of reproductive females give birth to single offspring ([30]; unpub. data). Births in my study area occur from mid June to late July, varying with spring weather conditions and the use of torpor by females [30], [31]. Embryonic development and parturition in bats are delayed when females use torpor due to inclement weather and reduced prey availability [32], [33]. The mating system of *E. fuscus* is polygynous [34]. Males and females congregate at hibernation sites in late summer and mating takes place there in autumn and winter [26]. However, as in many other hibernating bats, ovulation and fertilization do not occur until spring when females leave hibernation [35]. Polyovulation occurs in *E. fuscus*, with between two and five ova released per ovary [36]. Resorption of excess embryos takes place during gestation [36]. At birth and at fledging, males and females are no different in mass or forearm length (a standard measure of body size for bats) [30], [37]. There is little information regarding the age of sexual maturity in male and female *E. fuscus*. Some, but not all yearling females give birth ([28]–[30], [38]; this study). In my study area in late summer, only one of 14 juvenile male *E. fuscus* had extended epididymes, evidence of spermatogenesis (J. Coleman, pers. comm.), although at a much more southern location (Maryland, USA), at least some males underwent spermatogenesis in the summer of their birth [38]. Juvenile male *E. fuscus* disperse, and maternity colonies rarely contain adult males, making estimation of the survival of male offspring difficult. However, female pups return to their natal colony to reproduce; in my study, no female pups were ever captured at a colony other than the one they were born in. Survival of juvenile females can thus be estimated through recapture at colonies. As with other species of bats [39], *E. fuscus* is long lived [40]; the record at my study site is 19 years (unpub. data).

My study took place at three maternity roosts of *E. fuscus* located in old school-buildings in Medicine Hat, Alberta (50°02′N, 110°40′W). Field work took place during the summers of 1990–1997 and 1999–2004. Each colony consisted of 50 to 100 adult females plus their offspring. Sample size varied depending on the analysis. For example, some analyses used only known mother-offspring pairs and not all pups were captured with their mothers.

Adult and volant juvenile *E. fuscus* were captured as they exited the roosts in the evening using mist nets, or inside the roosts during the day by hand. To minimize disturbance, non-volant juveniles were captured by hand at night while adult females were foraging. Individuals were identified by sex and reproductive condition. Adult females were categorized as pregnant (assessed via palpation of the abdomen), lactating (indicated by expression of milk), or post-lactating (indicated by a bare patch around the nipples but no milk expression; [41]). I categorized adult females as non-reproductive if there was no evidence of pregnancy or lactation, and the date of capture was after the first lactating females had been captured, assuming that by that time, pregnancy would be detectable. Females that were caught before that date, and were not obviously pregnant or lactating, were categorized as unknown regarding their reproductive status. Each individual was banded on the forearm with numbered plastic split rings for later identification, and was released at its place of capture.

To estimate age of juveniles, forearm length was measured to the nearest 0.1 mm using callipers. For older juveniles, the size of the epiphyseal gap was also measured, as it can be used to estimate age [30], [42]. Birth date of juveniles was estimated using a combination of forearm length and epiphyseal gap, and growth formulae developed using data from known-aged *E. fuscus* at my study site [30]. For adults, I estimated relative age via the amount of wear of the upper canines (see also [38], [43]). I placed adults into one of seven tooth-wear categories, tooth code 1 being the youngest individuals, with the least amount of tooth wear [30], [44]. Of 38 known-aged adults (i.e. banded as pups) recorded as having a tooth code of 1, all but two (94.7%) were one year old. I thus considered individuals with a tooth code of 1 to be yearlings. I estimated female pup survival based on recapture of banded individuals in years after birth.

## Results

A total of 899 juvenile and 672 adult *E. fuscus* were captured and banded during the study. Estimated birth date varied considerably within and among years; the earliest birth was 13 June while the latest was 4 August. The median birth date ranged from 20 June in 2001 to 14 July in 2002 (Table 1). Within years and colonies, births occurred over a period ranging from 19 to 31 days.

**Table 1:** Year to year variation in median birth date (ordinal date) and range of birth dates for *Eptesicus fuscus* at three colonies in Medicine Hat, Alberta.

Year	Colony	Median Birth Date	Range
1990	ESS	187	180–206
1990	MSS	189	177–201
1991	ESS	176	172–190
1991	MSS	178	171–191
1992	ESS	177	166–193
1992	MSS	175	165–185
1993	ESS	172	166–193
1993	MSS	175	167–187
1994	ESS	175	164–194
1994	MSS	175	168–183
1995	ESS	181	173–196
1995	MSS	180	172–193
2001	ESS	171	164–186
2002	ESS	195	187–216
2002	CONN	190	178–211
2003	ESS	176	168–189
2003	CONN	177	169–186

**Figure 1:**
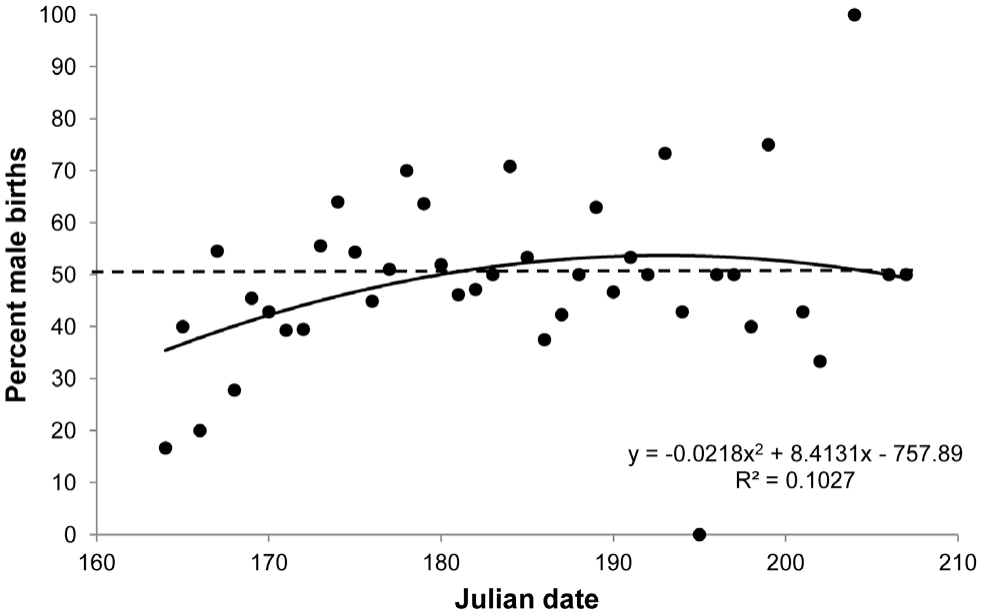
Seasonal variation in offspring sex ratio. Pattern of variation in offspring sex-ratio of *Eptesicus fuscus* with birth date at three colonies in Medicine Hat, Alberta, Canada from 1990 to 2004.

The overall sex ratio of pups was not significantly different from 1 (461 males, 438 females; 2-tailed binomial test, p = 0.46). In addition, no year had an overall sex ratio different from 1 (n = 10 years), and no individual colony had a sex ratio different from 1 in any particular year (n = 15 colony-years). However, sex ratio varied significantly with birth date (Fig. 1). The sex ratio of pups born on or before 22 June was significantly female biased (39.7% males; n = 150; p = 0.007), while pups born after 22 June were slightly but significantly male biased (53.8%; n = 749; p = 0.041). Early births were only female-biased in years when the parturition period was early. For example, in the three years when births were particularly late (1990, 1995 and 2002; Table 1), early births (during the first 9 days of the parturition period) were equally male and female (50.0% male, n = 74), and the overall sex ratio was also not different from 1 (50.3%, n = 362).

**Figure 2:**
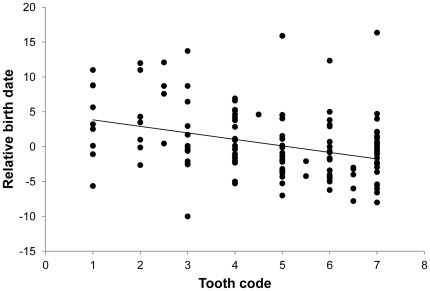
Correlation between age of females and parturition date. Relationship between age of female *E. fuscus* (as indicated by tooth wear), and the date they gave birth relative to the median date of birth for that year and colony, at three colonies in Medicine Hat, Alberta, Canada.

Parturition date of individual females was influenced by their age, as assessed by tooth wear. Within a year, older females gave birth earlier than younger females did (Fig. 2; r^2^ = 0.118, df = 135, t = 4.25, p<0.0001). Of females known to have given birth on or before 22 June (n = 16), none had a tooth code less than 4, and only two had a code of 4. Of those giving birth after 22 June (n = 136), 52.2% had a tooth code of 4 or less, including 13 with a tooth code of one. There was a significant difference in tooth code distributions between females giving birth on or before 22 June versus after it (χ^2^ = 7.52, df = 1, p = 0.006). Despite this relationship, the proportion of male pups was not influenced by relative age of the mothers (r^2^ = 0.04, df = 5, t = 0.46, p = 0.67). Females also did not differ in body size between those that gave birth to males (mean forearm length 47.46+/−1.61 mm) and those that gave birth to females (47.27+/−1.69 mm; t = 0.73, df = 146, p = 0.47).

**Figure 3:**
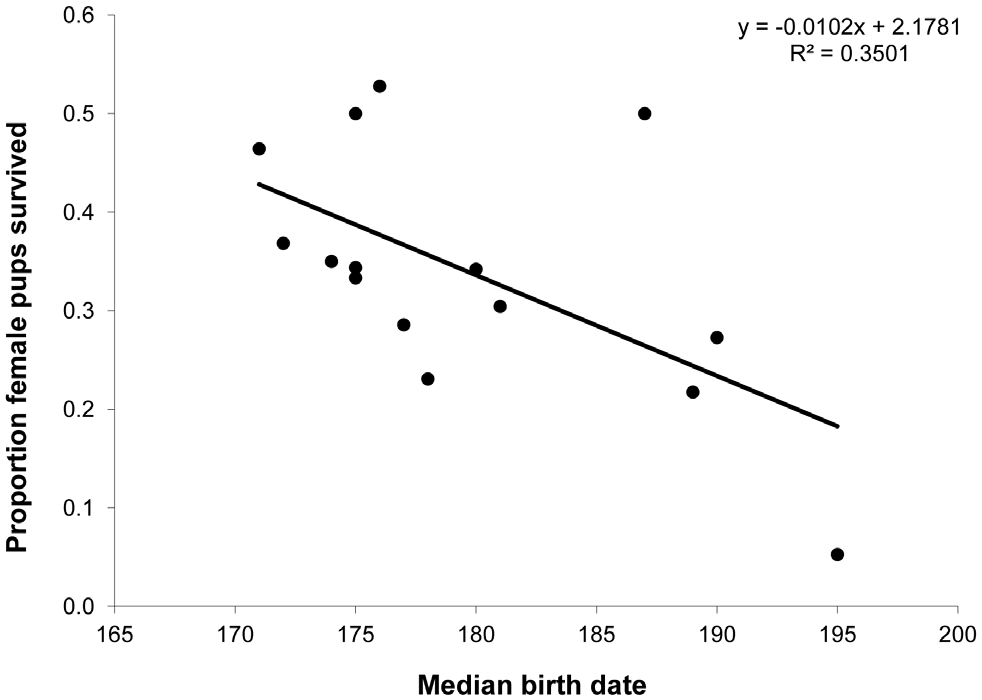
Correlation between pup survival and median birth date. Relationship between the median birth date for a colony of *E. fuscus* in a specific year, and the proportion of juvenile females known to have survived over their first winter.

Known survival of banded female pups varied among years. The later the median birth date was for the colony, the lower the survival rate (Fig. 3; t = 2.65, df = 13, p = 0.020).

Not all females in the colonies gave birth each year, and the proportion of reproductives varied with female age. For example, in 1990, a late parturition year, before there were any banded, known-aged individuals, females with the least tooth wear (tooth code 1), had a significantly lower rate of reproduction (52.2%, n = 23) than did females with greater tooth wear (categories 2–7; 92.0%, n = 87; 2-tailed Fisher Exact test, p<0.001). In 1991, an early parturition year, most known first-year females were reproductive (85.7%, n = 14), as were older females (94.1%, n = 84), and the difference was not significant (p = 0.59). Combining all years, known aged one-year old females and those with tooth code 1 were significantly less likely to reproduce (69.1%, n = 97) than were older individuals (96.1%, n = 534; χ^2^ = 76.9, p<0.001).

Data for female pups that returned and reproduced as one-year olds were limited because of the relatively low pup survival-rate and the need to capture banded one-year olds at a time when reproductive status could be assessed. None-the-less, the data indicate that reproduction by female pups was influenced by their date of birth. For pups born in 1991 and 1994, the two years with early parturition and sufficient subsequent data, 30.8% of pups born on or before 22 June survived and reproduced as one-year olds (n = 26), while only 6.6% of pups born later, returned as one-year olds and were reproductive (n = 61). This was a significant difference in reproductive rate (Fisher Exact test, p = 0.005).

## Discussion

My results, in conjunction with aspects of the biology of *E. fuscus*, support the hypothesis that adaptive, seasonal manipulation of offspring sex-ratio occurs due to unequal benefits provided by producing a daughter or a son early in the reproductive season. My results also indicate, however, that seasonally-skewed offspring sex ratios only occur in years when the overall parturition period is early. In other words, selection has favoured some control of offspring sex-ratio by females only when the benefit of early production of female offspring is large. This suggests not only the ability by female *E. fuscus* to influence the sex of their offspring, but also a finely-tuned response to environmental conditions and time of year.

With few exceptions [45], [46], previous reports of offspring sex ratio in bats found an overall 1∶1 ratio (e.g. [47]–[53]). However, most studies did not examine whether sex ratio varied seasonally, or with female age or body condition. Only one study reported a seasonally-skewed ratio [50]. *Myotis yumanensis*, a hibernating bat breeding at a similar latitude to my study area, produced a female-biased sex ratio late in the breeding season, and older females gave birth earlier than younger females. The results were interpreted in the context of the Trivers and Willard [6] hypothesis. Whether a consistent or variable pattern of seasonal sex-ratios occurred was not investigated.

In my study, the overall sex ratio of *E. fuscus* offspring was not different from 1∶1, as also found in previous studies of this species [37], [47], [54]. However, in years when environmental conditions allowed early births, the earliest births were biased almost 2∶1 in favour of females. There are a number of possible explanations for this, although several are not supported by the data. For example, as no sex-ratio bias occurred in years with late parturition, the seasonal skew did not result simply from a difference in embryonic development time between the sexes.

Survival of female *E. fuscus* over their first winter was higher for early-born females, as has also been reported for another hibernating bat, *Myotis lucifugus* [55]. The same should be true for males, however. Early birth provides juveniles with a longer period in which to grow and accumulate fat reserves required to successfully hibernate over the winter. Thus, I suggest that the survival benefit of early birth applies to both sexes and can not explain the skewed sex ratio early in the reproductive period.

Offspring sex-ratios that seasonally stray from Fisher's [5] expected 1∶1 ratio (e.g. [8], [13]–[20]), have been explained in two ways. Early born males may realize increased reproductive success due to the larger size or better body condition they can attain and the better competitive ability they have in polygynous breeding systems (the Trivers-Willard hypothesis; [8], [13], [15], [19]). In such species, it is argued that females benefit less from increased size or condition and thus a male-biased sex ratio is favoured early in the year. In other species, early births may benefit one sex more than the other by increasing that sex's probability of early sexual maturation and reproduction [9]. The result can be a female-biased sex-ratio early in the season [9], [21]. In either scenario, selection favours early-reproducing females that produce the sex that benefits the most from the longer growing season available to it. The inclusive fitness of such females is greater than that of females producing the other sex, and the result is an early season bias in offspring sex ratio.

My data do not support the Trivers-Willard hypothesis that early-born males benefit reproductively by being able to achieve larger size or better body condition. If this was the case, then an early-season, male-biased sex ratio would be predicted. I found the opposite bias. Although *E. fuscus* has a polygynous mating system [34], adult males are not larger than females [25] and there may be no reproductive advantage for males in having larger size.

My data support the early maturation hypothesis for sex-ratio variation in *E. fuscus*. Although overall, one-year old females reproduced at a significantly lower rate than did older females, and gave birth later in the season, female pups born early had a higher reproductive rate as one-year olds than did later-born pups. This is also true for *M. lucifugus* [55]. Thus, giving birth to females early in the season increases the inclusive fitness of females through more rapid reproduction of their daughters. The question is, why does the same not apply to juvenile males?

I suggest that an unusual aspect of the reproductive biology of hibernating bats, delayed ovulation, results in different probabilities of early maturation for male and female offspring, and favours production of females early in the year. Mating and ovulation in these species are temporally separated. Mating takes place during the autumn and winter at hibernation sites [56], while ovulation does not occur until spring when the females leave hibernation [35]. Thus, males undergo spermatogenesis during the summer, when conditions are favourable for this expensive process, while females do not ovulate until the following spring, and the costs of gestation and lactation occur a year after the costs of reproduction occurred in males. In the short growing season of northern latitudes, juvenile males may simply not have the time or resources to undergo spermatogenesis while also completing growth and accumulating fat reserves for hibernation. Indeed, in many temperate-zone hibernating bats, males do not reach sexual maturity until their second summer, while at least some one-year old females give birth (e.g. [57], [58]). In my study area, there is little evidence of sexual maturity in juvenile males, and I suggest the short season precludes completion of spermatogenesis. If this is the case, then early birth would have little or no effect on male age at maturity, and mothers giving birth early would benefit most by giving birth to females, as they have the opportunity to reproduce at one-year old, a year earlier than male offspring.

I predict there is complex geographic variation in seasonal sex-ratio patterns, related to the length of the growing season. In areas with a longer season than my study area, juvenile males may have a higher probability of reaching sexual maturity prior to the mating season in the autumn. This is apparently the case in Maryland [38], considerably further south than my site. In such areas, the benefit of producing a son or a daughter early in the season may be similar and no seasonal sex-ratio bias would be expected. In areas with an even shorter season than my study area, the probability of juvenile females reaching maturity in their first year may be as low as that of males. Again, no sex-ratio bias would be favoured. Although the evolution of such variation in sex-ratio patterns might be limited if gene flow occurs among populations, morphological traits of *E. fuscus* vary geographically [59], and a recent genetic study suggests that significant adaptive differentiation occurs among populations, despite male-mediated gene flow [60].

Adaptive variation in sex of offspring is possible in *E. fuscus* because females polyovulate [36]. More embryos implant than are eventually born, and resorption of excess embryos takes place during gestation [36]. Although the exact timing is unknown, this means that if selective resorption occurs based on sex of the embryos, as it does in some other mammals [61], it could occur relatively late, at a time when the relative date of birth has already largely been determined.

Most studies that report seasonal fluctuations in sex ratio do not reveal whether a consistent pattern occurs among years (but see [18]). This is despite the fact that year-to-year variation in environmental conditions results in different timing of reproduction and presumably therefore, different degrees of skewed benefit to males and females born at the beginning of the reproductive season. Indeed, in a few cases, sex-ratio skew did vary from year to year [1], [24]. The seasonally-biased sex ratio in *E. fuscus* also only occurred in years when parturition was relatively early. Few late-born female pups returned as one year olds and reproduced, suggesting that in years with late parturition, the fitness benefits of producing female or male offspring early in the season are similar.

Variation in the timing of parturition in *E. fuscus*, both among years and among individuals within a year, is clearly important for the evolution of adaptive sex-ratio manipulation in this species. Several factors combine to produce that variation in reproductive timing, and many or all of those factors are present in other hibernating bats, and in other mammals that hibernate. This suggests the likelihood that other species may also adjust sex ratio, given appropriate conditions and opportunities. Indeed, in European ground squirrels (*Spermophilus citellus*), individuals vary in time of emergence from hibernation and thus parturition date, and early-born litters are male biased [62].

Spring weather influences the timing of emergence from hibernation in mammals and thus the timing of mating, ovulation, and parturition (e.g. ground squirrels: [63]–[65]). In species, such as temperate-zone bats, that can enter daily torpor, gestation is also slowed by cold or wet weather and use of daily torpor [30], [33], [66], [67]. Variation in body condition (fat reserves) among females should lead to variation in use of daily torpor and thus parturition timing within a colony; females with greater fat reserves can afford to use less torpor and thereby benefit by giving birth earlier. In several species of hibernating bats, including *E. fuscus*, young-of-the-year females enter and exit hibernation at a lower mass than older females do [30], [55]. If this results in greater use of torpor during spring, it could explain the later birth dates for one-year old *E. fuscus* (this study), and *M. lucifugus* [55].

My results suggest that variation in body condition of female *E. fuscus*, resulting in differential use of torpor, influences timing of parturition and the benefits of producing a female offspring. If so, then as suggested for other species [1], [3], [12], [13], female body condition interacts with environmental variation to produce annual variation in sex-ratio adjustment. However, unlike most studies of mammalian sex-ratio adjustment, in which females in good condition preferentially produce sons [68], because of the effect of birth date on sexual maturity of females, *E. fuscus* in good condition produce female offspring in good (i.e. early parturition) years. Similar patterns of offspring sex-ratio variation should occur in other species of hibernating bats, if the opportunity for selecting sex of offspring exists. However, further complicating the pattern of sex ratios is the likelihood that season-length influences the relative benefit of one sex over the other. This should add spatial (geographic) variation to the temporal (annual, seasonal) variation seen in *E. fuscus* and other species. This complexity will only be clarified through other long-term studies on populations of a variety of species.
